# Association between Hearing Aid Use and Cognitive Function in Persons with Hearing Impairment Stratified by Cardiovascular Risk

**DOI:** 10.3390/jpm14050479

**Published:** 2024-04-29

**Authors:** Kouki Tomida, Sangyoon Lee, Keitaro Makino, Osamu Katayama, Kenji Harada, Masanori Morikawa, Ryo Yamaguchi, Chiharu Nishijima, Kazuya Fujii, Yuka Misu, Hiroyuki Shimada

**Affiliations:** Department of Preventive Gerontology, Center for Gerontology and Social Science, National Center for Geriatrics and Gerontology, Obu 474-8511, Aichi, Japan

**Keywords:** cardiovascular risk, cognitive function, hearing aid, hearing impairment, older adults

## Abstract

The purpose of this study was to conduct a cross-sectional analysis of the association between hearing aid use and cognitive decline in community-dwelling older adults with hearing impairment, stratified by cardiovascular risk level. This cross-sectional study covers 1857 hearing-impaired individuals selected among 10,674 community-dwelling older adults (≥65 years of age) in Japan. We investigate the association between hearing aid use and cognitive decline stratified by cardiovascular risk level, by assessing self-reported hearing impairment and hearing aid use, absolute cardiovascular risk, cognitive function, and potential confounding factors. The association between hearing impairment severity and increased cardiovascular risk, and the benefit of hearing aid use in preventing cognitive decline, were examined in a binomial logistic regression analysis, with the presence of cognitive decline as the objective variable. In the low cardiovascular risk group, hearing aid users had a lower odds ratio for decline in executive function than non-users (odds ratio = 0.61, 95% confidence interval: 0.39–0.98). However, there was no significant association between hearing aid use and cognitive decline in the high cardiovascular risk group (*p* > 0.05). Among older adults with hearing impairment, hearing aid use was associated with the maintenance of executive function in individuals of low cardiovascular risk.

## 1. Introduction

Age-related hearing impairment is the most common sensory impairment associated with aging, with an estimated 1.57 billion people worldwide with hearing impairment by 2019 [1]. This condition leads to increased disability-adjusted life years, which represent health losses [2]. Hearing impairment has been reported to cause communication difficulties, social isolation, and depression, and has been associated with cognitive decline and dementia [3,4]. A previous study reported that a decrease in the frequency of daily conversation is associated with an elevated risk of developing dementia [5]. The association between dementia and hearing impairment has received significant societal attention in recent years, as hearing impairment has been reported to be the most relevant of the modifiable risk factors for dementia [6]. Hearing impairment has also been reported to be associated with mild cognitive impairment [7]; thus, appropriate countermeasures should be taken as early as possible when people begin to notice hearing impairment.

Age-related hearing impairment is a sensorineural hearing impairment, characterized by damage to the areas that perceive sound [8]. The main cause is reported to be age-related damage to the hair cells in the cochlea, resulting in a decrease in their number and loss of auditory hairs [9]. Since hair cells are responsible for detecting and amplifying sound, it has been suggested that, when hair cells are damaged, they are unable to successfully transmit sound information to the brain, leading to cognitive decline [10]. In particular, atherosclerotic diseases such as hypertension, diabetes, and cardiovascular disease are thought to increase microcirculatory disturbances, chronic inflammation, and oxidative stress, which can damage the cochlea and auditory nerve and promote more severe hearing impairment [11]. It has also been reported that subclinical atherosclerosis in midlife is associated with hearing impairment in older adults, suggesting that the prevention and control of carotid atherosclerosis in midlife may have a positive impact on hearing health in older adults [12].

Currently, there is no curative treatment for age-related hearing impairment, and it has been reported that compensating for hearing impairment through the use of hearing aids can help prevent dementia and improve quality of life [13]. Recently, it has been shown that hearing impairment is associated with frailty [14], and preventing and controlling hearing impairment is becoming increasingly important in maintaining physical, mental, and cognitive function. In addition, a longitudinal study investigating the association between hearing impairment and physical function decline made the observation that hearing aid users have greater walking endurance [15]. Therefore, hearing aids can be an important tool for older adults with hearing impairment to maintain an active lifestyle. However, a nationwide survey in Japan suggests that hearing aids are not widely used in Japan compared to the United States and Europe, and that the benefits of hearing aids may not be maximized, because the introduction of hearing aids is considered only after the hearing impairment has become more severe [16]. As noted above, microcirculatory disturbances, such as those caused by cardiovascular disease, can damage the cochlea and auditory nerves and accelerate age-related hearing impairment [17]. In this context, the relationship between hearing aid use and cognitive function in the hearing impaired should be examined for individual conditions, especially in light of the impact of cardiovascular risk.

Therefore, the purpose of this study was to conduct a cross-sectional analysis of the association between hearing aid use and cognitive decline in community-dwelling older adults with hearing impairment, stratified by cardiovascular risk level. We hypothesized that, in Japan, where the introduction of hearing aids tends to be delayed, those at a higher risk for cardiovascular disease would have a more severe hearing impairment, which could reduce the benefits of hearing aid use.

## 2. Materials and Methods

### 2.1. Participants

This cross-sectional study involved community-dwelling older Japanese adults (≥65 years of age), who resided in Japan. They were recruited from a sub-cohort of a large cohort study, which aims to identify the risks of geriatric syndromes that occur with aging and to identify effective ways to treat them [18].

Individuals were interviewed regarding their self-report questionnaire assessment of hearing impairment and hearing aid use; those that were not hearing-impaired and those who had deficits in their assessment results were excluded. We then applied the following exclusion criteria: (1) a history of Alzheimer’s disease, Parkinson’s disease, or stroke; (2) impairment in basic activities of daily living (ADLs); (3) needing support and care by the long-term care insurance (LTCI) system at the time of assessment; (4) severe cognitive impairment (Mini-Mental State Examination [MMSE] score ≦ 21); and (5) missing data of exclusion criteria. Participants were evaluated by medical history assessments, sociodemographic information, blood sampling, and physical activity measurements.

The health check-up survey was undertaken by well-trained nurses and study assistants in community centers. All staff received training from the authors in terms of the protocols for administering the assessments prior to study commencement.

### 2.2. Ethical Approval

This study was conducted in accordance with the principles of the Declaration of Helsinki. The study protocol was approved by the Ethics Committee of the National Center for Geriatrics and Gerontology (approval number: 1440-5). Written informed consent was obtained from all participants before their inclusion in the study.

### 2.3. Measures

#### 2.3.1. Assessment of Self-Reported Hearing Impairment and Hearing Aid Use

Prior to the start of the study, the authors trained staff on the protocols for administering participant assessments. During these assessments, nurses asked participants whether they used hearing aids on a daily basis.

Participants also completed the Hearing Handicap Inventory for the Elderly Screening Version (HHIE-S) [19]. The HHIE-S questionnaire measures activity and participation restrictions caused by hearing impairment in various situations of daily life. The 10 items of the HHIE-S are classified into two subscales: one exploring emotional consequences, and another exploring social or situational effects. Each item has three response options: “yes” (score = 4), “sometimes” (2), and “no” (0). Hearing aid users and those with a total HHIE-S score greater than 8 were classified as having a hearing impairment [19].

#### 2.3.2. Estimation of Absolute Cardiovascular Risk

The revised WHO risk estimation chart was used to evaluate 10-year cardiovascular disease risk [20]. This chart shows the absolute risk of cardiovascular disease events according to an individual’s risk status, with higher risk scores indicating a greater burden of risk factors. The development group calibrated the prediction model for 21 regions of the world, with region-specific prediction charts. Two types of prediction tables are available: laboratory-based models that include medical history and blood data, and non-laboratory-based models consisting of convenient variables in resource-limited settings. In this study, we used a laboratory-based risk estimation model including age, sex, a history of diabetes, smoking status, systolic blood pressure, and total cholesterol for a high-income Asia–Pacific region, including Japan [20].

A current history of diabetes was assessed during a face-to-face interview with a nurse. The nurse measured systolic blood pressure using an automated blood pressure monitor, while the participant was seated. Serum total cholesterol levels (in mmol/L) were measured by the cholesterol oxidative enzymatic (COD-POD) method in a laboratory (SRL, Inc., Japan, Tokyo). Smoking status (smoked regularly, current vs. former/never) was assessed by the research assistants. Finally, we calculated absolute cardiovascular risk (%), based on the above risk status, using the revised WHO risk estimation charts and stratified cardiovascular risk levels, namely, low (≤20%) and high (≥20%).

#### 2.3.3. Measurements of Cognitive Function

Cognitive function was assessed using the National Center for Geriatrics and Gerontology–Functional Assessment Tool (NCGG-FAT) [21,22], which was administered by well-trained staff using appropriate testing protocols. The primary outcomes included four cognitive domain tests: memory (word list memory-I [immediate recognition], word list memory-II [delayed recognition]), attention (electronic tablet version of the Trail Making Test [TMT] Part A), executive function (electronic tablet version of the TMT Part B), and processing speed (electronic tablet version of the Symbolic Digit Substitution Test). The NCGG-FAT has been found to have high test–retest reliability and moderate-to-high validity among community-dwelling older adults [21]. All the tests have established standardized thresholds for defining objective cognitive impairment in the corresponding domains (a score of ≥1.5 standard deviations [SD] below the age- and education-specific means, based on an algorithm obtained from a database including over 10,000 community-dwelling older adults), which were derived from a population-based cohort [23].

#### 2.3.4. Potential Confounding Factors

Potential confounders of dementia and hearing impairment included age, gender, body mass index (BMI), years of education, and exercise habits [6]. The BMI was calculated using height and weight and measured using a bioelectrical impedance analyzer (Tanita MC780A; Tanita Corporation, Tokyo, Japan) [24]. To assess exercise habits, participants were asked, “Do you exercise moderately for your health?” and participants who answered “no” were considered physically inactive. Otherwise, depression was assessed using the 15-item Geriatric Depression Scale (GDS) [25]. A cutoff score of 6 or higher has an 82% sensitivity and 75% specificity in a structured clinical interview for depression [26]. Cognitive functioning was assessed using the Mini-Mental State Examination (MMSE) [27,28].

### 2.4. Statistical Analysis

Baseline characteristics were compared according to cardiovascular risk levels (low- and high-risk levels), using the unpaired *t*-test for continuous variables and a χ2 test for categorical variables.

To examine the association between hearing aid use and cognitive function, a binomial logistic regression analysis was performed in the presence or absence of functional decline in each cognitive domain as the objective variable. This model was applied to the overall sample and to the cardiovascular-risk-level-stratified sample (low and high risk). In these models, odds ratios (ORs) and 95% confidence intervals (CIs) for cognitive function were calculated in crude and adjusted models, using hearing aid use as the explanatory variable. Adjusted models were adjusted for the following potential confounding factors: age, sex, BMI, educational level, and exercise habits. Analyses were conducted using IBM SPSS Statistics software package version 27.0 (SPSS Corp., Armonk, NY, USA). The level of statistical significance was set a priori at *p* < 0.05.

## 3. Results

### 3.1. Participants

**Figure 1:**
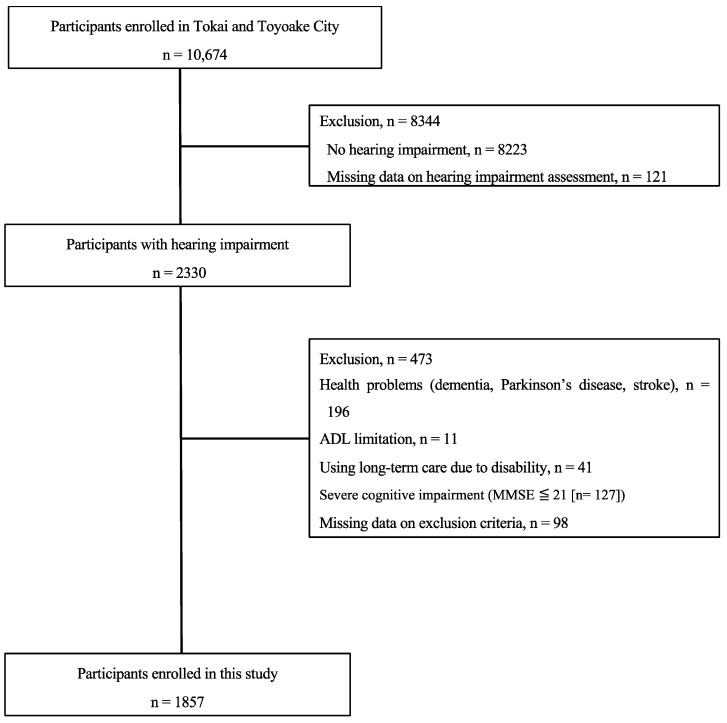
Flowchart of sample selection.

Among the 10,674 individuals that participated in the health check-up survey, 2330 were hearing-impaired and, therefore, pre-selected for the study. After exclusion criteria were applied, 1857 individuals were enrolled (Figure 1). Data for analysis were obtained from medical history and sociodemographic information, blood sampling, and physical activity measurements.

### 3.2. Characteristics of Hearing Impaired by Estimated Cardiovascular Risk Level

Among the 1857 participants with hearing impairment, 985 (53.0%) were allocated to the cardiovascular low-risk group and 872 (47.0%) were allocated to the high-risk group. The differences in participant characteristics between the two cardiovascular risk groups are shown in Table 1. Significant differences were found in all components of the WHO risk estimation model: age (*p* < 0.001), sex (*p* < 0.001), diabetes prevalence (*p* < 0.001), percentage of current smokers (*p* < 0.001), systolic blood pressure (*p* < 0.001), and total cholesterol (*p* < 0.001). In addition, there were significant differences between cardiovascular risk levels in BMI (*p* < 0.001), education level (*p* = 0.038), and hearing aid use (*p* = 0.014). There were no significant differences in history of depression, HHIE-S scores, MMSE scores, and exercise habits (*p* > 0.05).

### 3.3. Associations between Hearing Aid Use and Cognitive Function

Table 2 presents the results of the binomial logistic regression analysis for the total sample (n = 1857), the presence of a functional decline in each cognitive domain as the objective variable, and hearing aid use as the explanatory variable.

In both the crude and adjusted models, there was no significant association between hearing aid use and cognitive function for functional decline in each cognitive domain (memory, attention, executive function, and processing speed) (*p* > 0.05).

### 3.4. Associations between Hearing Aid Use and Cognitive Function Stratified by Cardiovascular Risk Levels

The results of the binomial logistic regression analysis in the cardiovascular-risk-level-stratified sample are shown in Table 3. In the low cardiovascular risk group (n = 985), there was an association between hearing aid use and cognitive function in persons with hearing impairment in the cognitive domain of executive function (OR, 0.61; 95% CI: 0.39–0.98; *p* = 0.039). However, no other statistically significant associations with cognitive function were detected. In the high cardiovascular risk group (n = 872), the association between hearing aid use and cognitive function in persons with hearing loss was not significant with any cognitive function (*p* > 0.05).

## 4. Discussion

This cross-sectional study used a logistic regression analysis with data from 1857 community-dwelling older adults with a hearing impairment, stratified by cardiovascular risk level, to determine associations between hearing aid use and the function of cognitive function. The result, a stratified analysis by cardiovascular risk level, showed no association between hearing aid use and the function of cognitive function in the high-risk group. This association, however, was found in the cardiovascular low-risk-level group. A possible explanation for this discrepancy is that high cardiovascular risk levels may adversely affect the progression of hearing impairment, possibly overriding the benefit of hearing aid use. As shown in Table 1, the group at higher cardiovascular risk has more people with diabetes, a smoking habit, higher blood pressure and total cholesterol levels, and a significantly higher BMI than the group at a lower cardiovascular risk. The number of hearing aid users is also significantly higher in the high cardiovascular risk group. The results emphasize the importance of introducing hearing aids before the hearing impairment becomes more severe and maintaining a lifestyle that keeps the level of cardiovascular risk low, in order to maximize the benefits of hearing aids.

Currently, there is no definitive cure for dementia, and attempts are being made to develop a variety of preventive interventions and treatments to delay its onset and progression. Hearing impairment has been reported as a relevant risk factor for dementia [6]. It has been suggested that hearing impairment limits the activities of daily living and narrows the life space [29] and may make it difficult to maintain an active lifestyle, which is important for dementia prevention [29]. Furthermore, it has been reported that the risk of nursing care is 1.7 times higher for those with hearing impairment, when accompanied by a feeling of loneliness [30]. In Japan, dementia is the leading cause of the need for nursing care [31], and appropriate care for hearing impairment may lead to the prevention of dementia and the need for nursing care. Indeed, early intervention with the use of hearing aids has been reported to prevent the onset of dementia and the progression of its symptoms [13]. However, the mechanisms linking hearing impairment and dementia have not been elucidated, and it remains unclear how hearing impairment affects various cognitive domains. This study showed an association between hearing aid use and a reduced decline in executive function in community-dwelling older adults with hearing impairment and low levels of cardiovascular risk. Executive function represents cognitive and intellectual abilities controlled by the frontal lobes, which are considered higher in the cognitive hierarchy than cognitive domains such as memory, perception, language, etc. [32]. Executive dysfunction has also been reported to be associated with depression, and the clinical features of depression and frontal lobe disorders are thought to be similar [33]. Age-related hearing impairment has been associated with depression and has been linked to cognitive decline and dementia [34]. Thus, in individuals with hearing impairment, it may promote a decline in executive function, which is regulated by frontal lobe function.

Age-related hearing impairment is a sensorineural hearing impairment caused by age-related damage to the outer hair cells of the cochlea and a decrease in the number of cells [34]. Recent evidence indicates that reactive oxygen species are involved in the development of hearing impairment [35]. Previous studies have shown that age-related hearing impairment is accelerated by microcirculatory disturbances, chronic inflammation, oxidative stress, and cardiovascular disease [11]. It has also been reported that subclinical atherosclerosis in midlife is associated with hearing impairment in older adults [12]. These findings suggest that, when cardiovascular risk is high, microcirculatory impairment, chronic inflammation, and oxidative stress accelerate the cell death of outer hair cells in the inner ear, the cause of age-related hearing impairment, resulting in a more severe hearing impairment. As an effective countermeasure for these problems, it has been suggested that increasing physical activity through exercise may promote antioxidant activity and help prevent hearing impairment [36]. However, sufficient evidence has not yet been obtained to verify whether exercise contributes to the improvement of hearing impairment or to the maintenance of hearing function. Therefore, the introduction of hearing aids is considered to be the first choice for hearing impairment, and it is increasingly important to establish the timing of their introduction and appropriate evaluation methods.

This study is a large-cohort study of community-dwelling older adults with hearing impairment. Although a number of cardiovascular risks have been reported to be associated with hearing impairment, their impact on hearing aid use has not been fully explored. Therefore, we consider this study to provide a baseline for further research. However, there are several limitations to this study. First, the participants’ hearing tests were based on self-report questionnaires and did not use objective measures. Also, because this is a cross-sectional study, it is unclear whether hearing aid use contributed to cognitive function in a causal manner. In addition, another limitation is the lack of information about when participants started using hearing aids, their compliance and degree of hearing impairment, the character of their hearing problems, and the duration of their hearing impairment. In future studies, we would like to add objective assessments to the hearing screening, and analyze the effects of hearing aid use on cognitive function longitudinally to examine the relationship between hearing impairment and cognitive function in more detail.

## 5. Conclusions

The association between hearing aid use and cognitive function, stratified by cardiovascular risk level, was investigated in a cross-sectional analysis of community-dwelling older adults with hearing impairment. Among older adults with a hearing impairment, hearing aid use was associated with the maintenance of executive function in individuals of low cardiovascular risk. Increased cardiovascular risk level may promote arteriosclerotic, and increase the severity of sensorineural, hearing impairment. These results suggest that it is important to consider hearing aid use even before hearing impairment becomes severe, to keep the cardiovascular disease risk level low and to maintain lifestyle habits that limit inner ear dysfunction. Further verification is required to determine what effect the use of hearing aids has on the health status of older adults, including the prevention of cognitive decline.

## Figures and Tables

**Table 1:** Characteristics of hearing-impaired participants stratified by estimated cardiovascular risk level.

		Overall n = 1857	Cardiovascular Risk Levels		*p*
			Low-Risk Group (<20%) n = 985 (53.0%)	High-Risk Group (≧20%) n = 872 (47.0%)	
Age	years	76.1 ± 6.0	75.1 ± 6.2	77.3 ± 5.6	<0.001 ^a^
Sex, female	n (%)	875 (47.1)	568 (57.7) ^c^	307 (35.2) ^d^	<0.001 ^b^
Diabetes mellitus, yes	n (%)	260 (14.0)	24 (2.4) ^d^	236 (27.1) ^c^	<0.001 ^b^
Smoking status, yes	n (%)	142 (7.6)	120 (12.2) ^c^	22 (2.5) ^d^	<0.001 ^b^
Systolic blood pressure	mmHg	145.1 ± 19.6	134.3 ± 15.6	157.3 ± 16.3	<0.001 ^a^
Total cholesterol	mmol/l	5.3 ± 0.9	5.2 ± 0.8	5.4 ± 0.9	<0.001 ^a^
BMI	point	23.1 ± 3.1	22.8 ± 3.2	23.4 ± 2.9	<0.001 ^a^
Depression, yes	n (%)	37 (2.0)	24 (2.4)	13 (1.5)	0.146 ^b^
Education level	years	11.5 ± 2.4	11.6 ± 2.4	11.3 ± 2.4	0.038 ^a^
MMSE	score	26.7 ± 2.3	26.8 ± 2.3	26.6 ± 2.3	0.059 ^a^
HHIE-S	score	16.8 ± 8.0	16.5 ± 7.9	17.2 ± 8.1	0.073 ^a^
Hearing aid use, yes	n (%)	454 (24.4)	218 (22.1) ^d^	236 (27.1) ^c^	0.014 ^b^
Exercise habits, yes	n (%)	563 (30.3)	306 (31.1)	257 (29.5)	0.456 ^b^

BMI, Body mass index; MMSE, Mini-Mental State Examination; HHIE-S, Hearing Handicap Inventory for the Elderly–Screening. Data are expressed as mean ± standard deviation or numbers (%). ^a^ = *p*-values reported from unpaired *t*-test. ^b^ = *p*-values obtained by Pearson’s chi-squared test. ^c^ = Statistically significant association by adjusted standardized residual > 1.96 [*p* < 0.05]. ^d^ = Statistically significant association by adjusted standardized residual < −1.96 [*p* < 0.05].

**Table 2:** Binomial logistic regression results showing associations between hearing aid use and cognitive function.

Cognitive Function	Variables	Crude Model		Adjusted Model	
		OR (95% CI)	*p*-Value	OR (95% CI)	*p*-Value
Memory (Score < 1.5SD), yes	Hearing aid user	0.88 (0.59–1.31)	0.526	0.94 (0.62–1.42)	0.771
	Age			0.98 (0.95–1.01)	0.180
	Sex, female			0.60 (0.42–0.85)	0.004
	BMI			1.00 (0.95–1.06)	0.954
	Education level			0.91 (0.84–0.98)	0.015
	Exercise habits			0.63 (0.42–0.95)	0.028
Attention (Time < 1.5SD), yes	Hearing aid user	0.92 (0.59–1.42)	0.691	0.94 (0.62–1.42)	0.601
	Age			1.01 (0.98–1.04)	0.537
	Sex, female			0.85 (0.59–1.24)	0.408
	BMI			1.00 (0.95–1.07)	0.906
	Education level			0.95 (0.87–1.03)	0.182
	Exercise habits, yes			0.59 (0.38–0.94)	0.025
Executive function (Time < 1.5SD), yes	Hearing aid user	0.93 (0.69–1.25)	0.615	0.76 (0.55–1.03)	0.075
	Age			1.07 (1.05–1.10)	<0.001
	Sex, female			1.15 (0.89–1.50)	0.279
	BMI			1.02 (0.98–1.06)	0.359
	Education level			0.88 (0.83–0.93)	<0.001
	Exercise habits, yes			0.65 (0.47–0.88)	0.006
Processing speed (Score < 1.5SD), yes	Hearing aid user	0.75 (0.37–1.51)	0.416	0.71 (0.35–1.46)	0.357
	Age			1.02 (0.97–1.07)	0.430
	Sex, female			0.90 (0.51–1.59)	0.718
	BMI			1.02 (0.93–1.11)	0.744
	Education level			0.90 (0.79–1.02)	0.092
	Exercise habits, yes			1.04 (0.56–1.93)	0.910

**Table 3:** Binomial logistic regression showing associations between hearing aid use and cognitive function, stratified by absolute cardiovascular risk levels.

Cognitive Function	Variables	Cardiovascular Risk Levels			
		Low-Risk Group (<20%)		High-Risk Group (≧20%)	
		OR (95% CI)	*p*-Value	OR (95% CI)	*p*-Value
Memory (Score < 1.5SD), yes	Hearing aid user	0.86 (0.49–1.54)	0.621	1.04 (0.57–1.89)	0.910
	Age	0.98 (0.94–1.01)	0.213	1.00 (0.95–1.05)	0.966
	Sex, female	0.59 (0.38–0.93)	0.024	0.48 (0.26–0.89)	0.019
	BMI	0.96 (0.89–1.03)	0.221	1.08 (0.99–1.18)	0.080
	Education level	0.90 (0.81–0.99)	0.038	0.92 (0.82–1.04)	0.168
	Exercise habits, yes	0.62 (0.36–1.06)	0.082	0.68 (0.36–1.30)	0.216
Attention (Time < 1.5SD), yes	Hearing aid user	0.87 (0.47–1.61)	0.653	0.93 (0.49–1.79)	0.831
	Age	1.02 (0.98–1.06)	0.400	1.01 (0.95–1.06)	0.860
	Sex, female	0.67 (0.40–1.10)	0.111	1.06 (0.59–1.89)	0.844
	BMI	1.00 (0.92–1.08)	0.937	1.02 (0.93–1.12)	0.735
	Education level	0.94 (0.84–1.05)	0.248	0.95 (0.83–1.08)	0.400
	Exercise habits, yes	0.69 (0.39–1.24)	0.219	0.49 (0.23–1.03)	0.058
Executive function (Time < 1.5SD), yes	Hearing aid user	0.61 (0.39–0.98)	0.039	0.93 (0.61–1.42)	0.740
	Age	1.08 (1.05–1.11)	<0.001	1.07 (1.03–1.11)	<0.001
	Sex, female	0.93 (0.65–1.34)	0.698	1.33 (0.91–1.95)	0.141
	BMI	1.02 (0.96–1.08)	0.526	1.02 (0.96–1.09)	0.515
	Education level	0.84 (0.77–0.91)	<0.001	0.92 (0.84–1.00)	0.047
	Exercise habits, yes	0.65 (0.42–0.99)	0.045	0.66 (0.42–1.04)	0.070
Processing speed (Score < 1.5SD), yes	Hearing aid user	0.67 (0.25–1.80)	0.424	0.81 (0.28–2.29)	0.688
	Age	1.04 (0.98–1.11)	0.205	1.00 (0.92–1.08)	0.898
	Sex, female	0.78 (0.37–1.70)	0.526	0.88 (0.35–2.19)	0.777
	BMI	0.94 (0.83–1.07)	0.338	1.12 (0.98–1.28)	0.108
	Education level	0.78 (0.65–0.93)	0.007	1.04 (0.87–1.25)	0.677
	Exercise habits, yes	1.48 (0.67–3.30)	0.337	0.68 (0.25–1.89)	0.464

## Data Availability

The datasets used and/or analyzed during the present study are available from the corresponding author upon reasonable request.
